# The Role of Fluorine-18-fluorodeoxyglucose Positron Emission Tomography in Assessing the Response to Neoadjuvant Treatment in Patients with Osteosarcoma

**DOI:** 10.1155/2012/870301

**Published:** 2012-09-13

**Authors:** Carmelo Caldarella, Marco Salsano, Maria Antonietta Isgrò, Giorgio Treglia

**Affiliations:** ^1^Department of Bioimaging and Radiological Sciences, Institute of Nuclear Medicine, Catholic University of the Sacred Heart, Largo Gemelli 8, 00168 Rome, Italy; ^2^Institute of Biochemistry and Clinical Biochemistry, Catholic University of the Sacred Heart, 00168 Rome, Italy

## Abstract

*Aim*. The objective of this study is to systematically review the role of positron emission tomography (PET) and PET/computed tomography (PET/CT) with fluorine-18-fluorodeoxyglucose (FDG) in assessing the response to neoadjuvant treatment in patients with osteosarcoma (OS). *Methods*. A comprehensive literature search of published studies through March 2012 in PubMed/MEDLINE, Embase, and Scopus databases regarding whole-body FDG-PET and FDG-PET/CT in patients with OS was performed. *Results*. Twenty-two studies have investigated the role of FDG-PET and FDG-PET/CT in the evaluation of response to neoadjuvant treatment with either chemotherapy or radiation therapy in patients with OS. The main findings of these studies are presented. *Conclusion*. FDG-PET or PET/CT seems to be sensitive and reliable diagnostic tools in the assessment of metabolic response to treatment in patients with OS, after baseline PET evaluation has been performed in advance. However, false positive findings due to inflammation in sites of tumoral response should be considered.

## 1. Introduction

Osteosarcoma (OS) is the most common primary malignant bone tumour in children and adolescents, with a peak of incidence at the age of 15–19 years [1]. OS is a tumour derived from primitive mesenchymal cells, originated from bone and rarely from soft tissue [2]. Although OS can occur in any bone, it is most common in the metaphyses of long bones: distal femur, proximal tibia, proximal humerus, and around the knee [3–5]. OS has a high tendency to metastatic spread: 80% of all metastases arise in the lungs (20% of them at initial diagnosis) but metastases can also develop in bone and rarely in lymph nodes [6–9]. The 5-year survival rate for OS patients with metastases is 20% compared to 65% for patients with localised disease [10].

Usually, the treatment scheme for patients with OS is comprised of preoperative chemotherapy, surgical removal of all detectable tumour sites, and/or local treatment, followed by postoperative chemotherapy. The prognosis for patients with metastatic disease or recurrent disease remains poor [11, 12].

In order to correctly evaluate patients with OS in staging and restaging, a variety of diagnostic imaging modalities may be used, such as radiography, computed tomography (CT), magnetic resonance imaging (MRI), and traditional nuclear medicine techniques (bone scintigraphy). Fluorine-18-fluorodeoxyglucose positron emission tomography (FDG-PET) has been successfully used to evaluate different malignant tumours, such as musculoskeletal tumours [13]. Tumour cells have a metabolic activity higher than normal cells and usually show an increased uptake of FDG, a glucose analogue. Like many other malignant tumours, OS has an increased rate of glycolysis and consequently demonstrate an increased uptake of FDG. Standardised uptake value (SUV) can be used as semiquantitative measure of the metabolic activity of a specific region of interest [14].

Through the use of hybrid devices, integrating the high sensitivity of FDG-PET with the high spatial resolution of computed tomography (CT), a better diagnostic accuracy of PET/CT than PET and CT alone in detecting malignant tumours, such as OS, can be achieved [13–15].

Several studies have shown the potential role of FDG-PET and PET/CT in the diagnosis of OS; however, a systematic review of published data about the role of these methods in assessing the response to neoadjuvant treatment in patients with OS was lacking. The purpose of this study is therefore to systematically review the role of FDG-PET and PET/CT in this setting.

## 2. Materials and Methods

### 2.1. Search Strategy

A comprehensive computer literature search of the PubMed/MEDLINE, Embase, and Scopus databases was conducted in order to find relevant published articles on the diagnostic accuracy of FDG-PET and FDG-PET/CT in patients with osteosarcoma (OS). We used a search algorithm based on a combination of the terms: (a) “Sarcoma” or “sarcomas” or “osteosarcoma” or “osteogenic sarcoma” or “bone sarcoma” or “bone sarcomas” or “pediatric tumors” or “pediatric tumours” or “pediatric sarcomas” or “pediatric sarcoma” or “childhood sarcomas” or “bone tumors” or “bone tumours” or “osseous sarcomas” or “skeletal sarcomas” or “skeletal sarcoma” or “musculoskeletal sarcomas” and (b) “positron emission tomography” or “PET.” No beginning date limit was used; the search was updated until March 2012. Only articles in English language were selected. To expand our search, references of the retrieved articles were also screened for additional studies.

### 2.2. Study Selection

Studies or subsets in studies investigating the role of FDG-PET or FDG-PET/CT in assessing the response to neoadjuvant treatment in patients with OS were eligible for inclusion. Review articles, editorials or letters, comments, conference proceedings, articles not in the field of interest of this review, and case reports were excluded from this review. Only those studies or subsets in studies that satisfied all of the following criteria were included in the systematic review: (1) FDG-PET or FDG-PET/CT performed in patients with OS; (2) treatment response assessed by FDG-PET and FDG-PET/CT.

Three researchers (GT, MS, and CC) independently reviewed the titles and abstracts of the retrieved articles, applying the selection criteria mentioned above. Articles were rejected if they were clearly ineligible. The same three researchers then independently reviewed the full-text version of the remaining articles to determine their eligibility for inclusion. 

### 2.3. Quality Assessment

Two independent reviewers evaluated the methodology of the selected studies using the Quality Assessment Tool for Diagnostic Accuracy Studies (QUADAS) [16]. This 14-items tool is composed by five items related to verification bias, three items related to review bias, two items relating to generalizability and context and spectrum bias, and four to reporting. Reviewers, who were blinded to the purposes of the systematic review, recorded a score of “1” for “yes” and “0” for “no” for each of the 14 items; all disagreements were resolved by means of consensus. Interrater reliability was also evaluated.

## 3. Literature Data

Twenty-two studies have investigated the role of FDG-PET and FDG-PET/CT in assessing the response to neoadjuvant treatment with either chemotherapy or radiation therapy in patients with OS. A prospective design was employed in 10 studies [17–26], while the remaining 12 studies were conducted retrospectively [11, 27–37]. General characteristics of selected studies are summarized in Tables 1 and 2.

### 3.1. Quality Assessment

Overall, the studies included in systematic review have shown moderate methodological quality according to QUADAS [16]. Studies scored between 8/14 and 12/14 with a median score of 10/14. The index test and the reference standard were often interpreted without blinding, and this represents the most critical issue about the methodological quality of the included studies. Interrater reliability was substantial (kappa = 0.8).

### 3.2. Prospective Studies

The usefulness of FDG-PET in assessing the response to neoadjuvant treatment in OS patients was first investigated in 1996 by Jones et al. [17]. Nine patients with histological fine-needle confirmation of musculoskeletal sarcoma (including three OS patients) underwent FDG-PET and MRI before therapy (baseline study), after completion of one course of chemotherapy or combined radiation therapy/hyperthermia (early study) and after completion of neoadjuvant chemotherapy, shortly before surgical resection (posttherapy study). SUV and tumour to background ratio (TBR) of the lesions were obtained. Posttherapy FDG-PET studies demonstrated a generalized reduction of SUV and TBR values if compared with pretherapy studies. Moreover, this reduction was noticeable also in early studies and well correlated with histological evidence of extensive necrosis (at least 90% non-viable cells within the tumour after treatment). An increase in signal intensity on T2-weighted MRI images was observed after treatment, but it was not specific for necrosis. 

Three years later, Schulte et al. [18] investigated the usefulness of FDG-PET in assessing the response to neoadjuvant treatment in a wider population of patients with OS. Twenty-seven patients with histologically ascertained high-grade OS were examined before biopsy and after neoadjuvant chemotherapy. Tumour regression was assessed on 25 patients within the resected specimen. TBR of each lesion was measured both before (TBR1) and after (TBR2) chemotherapy, and TBR-ratio (TBR2/TBR1) was calculated thereafter. While no significant differences in TBR1 values were observed between responder and nonresponder patients, TBR2 was significantly lower in responder patients; TBR-ratio showed a close correlation to the histological extent of tumour destruction and could be used (cut-off level 0.6) to reliably distinguish between responder and nonresponder patients.

Ye et al. [19], in 2008, investigated the role of FDG-PET in the evaluation of response to neoadjuvant treatment in 15 patients with OS, when compared with histologically assessed necrosis (according to Salzer-Kuntschik's criteria). Eight patients had good chemotherapy responses, showing less than 10% area of viable tumour cells; seven patients had unfavourable chemotherapy responses. A moderate positive correlation was found between SUV-ratio (posttherapy SUV/pretherapy SUV) and histological degree of necrosis; a much stronger correlation was demonstrated between histological necrosis and TBR-ratio or posttherapy TBR. Particularly, patients with TBR-ratio < 0.46 could be reliably considered as good responders (in fact, all eight good responder patients showed TBR-ratios lower than 0.46), while patients with TBR-ratio > 0.49 could be reliably considered as poor responders (in fact, all seven poor responder patients showed TBR-ratios above 0.49). Instead, posttherapy SUV value was not related to histological outcome.

In the same year, Sato et al. [20] evaluated 13 patients with OS and investigated the relationship between FDG uptake in the tumoral tissue and expression of autocrine motility factor/phosphoglucose isomerase (AMF/PGI), a metastasis-related glycolytic enzyme induced by hypoxia, evaluated through immunohistochemical staining. The correlation between SUV values and disease progression was also investigated. While pretherapy SUV values were similar between patients who were completely free of disease and those with disease progression, higher posttherapy SUV values were related to disease progression and presence of distant metastases: particularly, posttherapy SUV > 5 was strongly related to poor overall survival and high risk to die of disease. Furthermore, posttherapy SUV correlated with expression of AMF/PGI, indicating that a higher FDG uptake is related to high glycolytic activity, which may be responsible for metastatic ability.

Benz et al. [21] evaluated the interobserver variability of parameters used to quantify FDG uptake (such as maximum SUV, peak SUV, mean SUV and TBR) in 33 patients with high grade sarcomas who underwent FDG-PET examinations before and after neoadjuvant chemotherapy (8 OS patients). Histological response was assessed by quantifying the degree of tumour necrosis. A decrease of FDG uptake parameters was observed in posttherapy scans, in comparison with baseline: particularly, changes in maximum SUV, peak SUV and mean SUV were significantly more pronounced in responders than in nonresponders. However, a higher interobserver variability was found in peak SUV, mean SUV, and TBR changes from baseline to posttherapy study than did individual baseline or posttherapy corresponding values. Maximum SUV showed absolute interobserver concordance in both baseline and posttherapy examinations, while peak SUV showed lower interobserver than did mean SUV and TBR. Therefore, they demonstrated that maximum and peak SUV, either with manual or semiautomatic delineation of tumour volume, provide the most robust measurement of glycolytic activity in sarcomas.

Two years later, the same authors investigated whether the adoption of a definite cut-off value for the reduction of metabolic activity could accurately predict the histological response in 12 patients with high-grade resectable sarcomas [22]. Particularly, 6 patients with OS were evaluated at baseline and after neoadjuvant therapy with FDG-PET/CT examination. A reduction in tumour maximum SUV > 60% was adopted to discriminate good metabolic responses; a degree of necrosis >90% within the tumour was used to discriminate good histological responses. They found that reduction of tumour FDG uptake was greater in histological responders than in nonresponders, showing a strong correlation with percent necrosis in the excised tumour, while changes in tumour size did not. Patients with OS and evidence of increase in tumour size and metabolic activity after therapy had 10% necrosis in the excised tumour and poor prognosis. A reduction in tumour maximum SUV > 60% correlated with histological necrosis in 5 of 6 patients with OS: the single metabolic “false negative” result (a patient with apparently no metabolic response but extensive histological necrosis) was presumably related to the low FDG uptake at baseline examination.

Denecke et al. [23] prospectively evaluated 27 paediatric patients with histologically proven sarcoma (11 OS and 16 Ewing sarcoma (ES)) to assess the response to chemotherapy with FDG-PET, in comparison with CT/MRI volumetry. All patients had the lesions surgically resected after neoadjuvant chemotherapy and all showed a reduction of maximum SUV values from baseline to postchemotherapy scan. However, a higher reduction was seen in ES, rather than in OS, patients; besides, responder OS patients showed a significantly higher reduction in maximum SUV and a lower posttherapy maximum SUV, while no significant differences were seen between responder and nonresponder ES patients. Threshold values of 2.8 and 0.62 for posttherapy maximum SUV and reduction in maximum SUV, respectively, reliably discriminated response from non-response in OS patients. Neither reduction in tumour volume, nor changes in T2 signal at MRI, nor soft tissue component reduction, nor baseline FDG uptake was useful predictors of favourable response to chemotherapy.

Forty-two patients with histologically proven high-grade sarcoma were studied by Tateishi et al. [24] by using MRI and FDG-PET/CT examination before (baseline), after the first cycle of chemotherapy (early), and after completion of neoadjuvant treatment (posttherapy) to predict histological response, overall and progression-free survival, and to correlate them with metabolic response as assessed by SUV ratio. Baseline, early and posttherapy SUV values were not statistically different between metabolic responders and nonresponders; conversely, early and posttherapy SUV, as well as percent SUV reduction values, were higher in histopathological nonresponders, while no correlation was found between histological necrosis and changes in tumour size. Higher percent SUV reduction values (greater than 35% between baseline/early and early/posttherapy) were related to higher likelihood of extensive necrosis on surgically resected tumour, longer progression-free and overall survival, while changes in tumoral size did not.

Bajpai et al. [25] evaluated the correlation between baseline and posttherapy FDG-PET/CT metabolic assessment of tumoral lesion and histopathological necrosis in 31 treatment naïve patients with OS who underwent neoadjuvant chemotherapy and surgery. Tumour volume, baseline, and posttherapy SUV, SUV ratio (posttherapy SUV/baseline SUV) and metabolic burden (MB : SUV × tumour volume) were available. A reduction in SUV and MB values was observed from baseline to postchemotherapy examination. All quantitative parameters, except baseline SUV, significantly correlated with histological necrosis. Particularly, 82% of patients were correctly identified as responders or nonresponders by using only SUV ratio, with a better prediction of response when cut-off value was set at 0.48. Furthermore, even if changes in tumour volume and posttherapy volume did not correlate with histological outcome, baseline tumour volume and SUV ratio were identified as independent predictors of histological necrosis. Baseline volume <300 mL and SUV ratio <0.48 identified 83% responder patients, when both were present. High variability of postchemotherapy maximum SUV values was observed within the responders group: it was ascribed to the fact that inflammation and reactive fibrosis in sites of tumoral response to chemotherapy may accumulate FDG as well.

Lastly, Im et al. [26] extensively studied the utility of volumetric and metabolic FDG-PET/CT indices after one course and after completion of chemotherapy in 20 patients with OS. Fourteen patients were enrolled prospectively and mostly received an addition scan between the first and second course, while 6 patients were recruited retrospectively. Volumetric and metabolic indices of prechemotherapy and postchemotherapy FDG-PET/CT examinations, such as metabolic tumour volume (MTV), total lesion glycolysis (TLG), maximum SUV, and their corresponding ratios (rMTV, rTLG, and rSUV), were calculated and compared with histological necrosis and the expression of glucose transporters (investigated by immunohistochemical staining). A good correlation was found between prechemotherapy MTV and tumour volume measured by means MRI; a much stronger correlation existed between histological necrosis and MTV, TLG, SUV, rMTV, and rTLG values. Therefore, histological response was reliably predicted by volumetric and metabolic FDG-PET/CT indices: particularly, a postchemotherapy SUV cut-off value of 3 showed absolute sensitivity and negative predictive value in predicting response to therapy in prospectively evaluated patients.

### 3.3. Retrospective Studies

In 2000, Nair et al. [27] retrospectively evaluated the results of serial FDG-PET scans of 16 untreated patients with OS, who underwent neoadjuvant chemotherapy using carboplatin and ifosfamide. Both visual qualitative scoring and semiquantitative evaluation (baseline and posttherapy TBR, and percent change in TBR) were performed and compared to histological necrosis. Visual assessment and posttherapy TBR value correctly predicted good histological response in all but one patient; instead, percent change in TBR showed high variability in histologically good responder patients, but no significant differences were seen between responder and nonresponder groups. FDG uptake in the site of a healing normal bone after successful treatment was hypothesized to explain incorrect prediction of histological necrosis by both visual and TBR assessment in one patient.

In the same year, Franzius et al. [28] studied 11 OS patients before and after chemotherapy. Tumour to nontumour (T : NT) ratios of baseline and postchemotherapy FDG-PET examinations were used to quantify the response to therapy in the primary tumour site and were correlated to bone scintigraphy T : NT ratios and histological necrosis (according to Salzer-Kuntschik's grading). Possible distant bone and soft tissue metastases were also assessed. No correlation was found between FDG-PET and bone scintigraphy T : NT ratios; conversely, all the patients with histological good response showed a decreasing FDG-PET T : NT ratios greater than 30% (median 65.5%) and a patient with poor response had an increasing FDG-PET T : NT ratio. Change in T : NT ratio from baseline to postchemotherapy bone scintigraphy was poorly related to histological outcome; besides, a reduction greater than 30% (median 26.5%) was infrequently observed. Seven bone metastases were assessed by FDG-PET and showed decreasing FDG uptake after therapy: three of them were not detected by bone scan. The authors argue that bone scintigraphy may reflect the healing response of bone in a site of successful treatment of lesions, rather than the presence of viable tumoral cells, as FDG-PET does.

Hawkins et al. [29] reviewed their experience on FDG-PET and neoadjuvant chemotherapy response in 33 children with bone sarcomas: 18 OS and 15 ES patients. Histological necrosis was assessed after surgical resection of the primary tumour. Significantly greater values of postchemotherapy SUV, SUV ratio (postchemotherapy SUV : prechemotherapy SUV) and lower percent necrosis on surgical specimens were observed in OS than in ES patients. Moreover, a significant correlation between postchemotherapy SUV, SUV ratio, and histological necrosis was found in OS patients: lower postchemotherapy SUV and SUV ratio values were associated with favourable response. However, correlation with progression-free survival and overall survival was not possible, because of short time followup.

Iagaru et al. [30] have investigated 14 patients with diagnosis of osseous and soft tissue sarcoma (7 OS patients), who underwent 2 consecutive FDG-PET examinations to evaluate chemotherapy response. Semiquantitative measurements of FDG uptake and response to therapy, as assessed according to the European Organization for Research and Treatment of Cancer (EORTC) criteria for PET, were compared with the histologically evaluated amount of tumour necrosis. In 57% cases (8 patients), a concordance was found between EORTC classification of patients by using baseline and posttherapy FDG-PET and histologically proven tumour necrosis. Discrepancies were partly explained as a consequence of increased FDG uptake in sites of chemotherapy-induced inflammation, whereas time interval from the end of therapy and posttherapy FDG-PET examination did not significantly differ between concordant and discordant patients.

In the same year, Mahajan et al. [31] evaluated 39 high-risk OS patients who underwent radiation therapy. Plain X-rays, CT, FDG-PET/CT, MRI, and nuclear bone scan were used for pretherapy and posttherapy assessment. Forty sites of irradiation were imaged with FDG-PET at baseline and post-irradiation: 72% of tumour sites showed significant decrease in FDG uptake, assessed by SUV, and after irradiation, 10 patients were metabolically stable, while one patient worsened from baseline to post-irradiation FDG-PET scan. Conventional anatomical imaging modalities (CT and MRI) showed stable disease in all patients, although the pattern of MRI enhancement within the lesions became more heterogeneous after irradiation. Both bone scan and FDG-PET showed nonavid lesions (despite apparently stable disease on anatomical imaging) after therapy in patients with evident clinical improvement. 

Costelloe et al. [32] have evaluated whether maximum SUV or TLG, by using FDG-PET/CT, was useful predictors of tumour necrosis and overall survival after initial chemotherapy in 31 consecutive patients with OS, before surgical resection of the primary tumour. Postchemotherapy maximum SUV and change in maximum SUV from baseline to postchemotherapy showed a significant correlation with progression-free survival and the degree of histological necrosis. Higher TLG values after chemotherapy and increasing percent TLG from baseline to postchemotherapy scan were associated with worse progression-free survival, while higher baseline TLG values were associated with poor overall survival and, weakly, with histological necrosis. 

Piperkova et al. [33] have retrospectively reviewed 93 patients with bone and soft tissue sarcomas to assess the usefulness of FDG-PET/CT for staging, restaging, and evaluation of response to treatment, focusing on the discrepancies between PET and CT examinations. Eighty-three studies were available to evaluate the response to treatment: concordance between PET and CT was found in 64 studies (77%), mostly with complete response or stable disease. In discordant studies, PET usually showed metabolic response, while stable disease or progression was observed on CT scan; moreover, in 5 discordant studies with more than 75% decrease in maximum SUV but no changes on CT scan, negativity on FDG-PET/CT was observed after 6 months of followup.

Hamada et al. [34] studied 11 patients with histological diagnosis of high-grade OS, determined from the open biopsy materials. Patients underwent neoadjuvant chemotherapy, then repeated morphological (MRI) and morphofunctional (FDG-PET) imaging before surgery. In 5 patients with histological evidence of good response (>90% necrosis), posttherapy scan SUV was lower than in 6 patients with poor histological response (<90% necrosis), despite no significant changes in tumour size were evident at MRI in both groups. Particularly, positive and negative predictive values of 100% for the identification of good and poor responses were obtained for SUV values < 2.5. A greater reduction in SUV from baseline to posttherapy examination well correlated with a better histological response: positive predictive value of 80% and negative predictive value of 100% were obtained when a cut-off value of 0.5 was set for SUV ratio.

Hawkins et al. [35] investigated the role of metabolic response after chemotherapy, as assessed by FDG-PET, in predicting outcome of 40 paediatric and young adult patients with extremity OS. Not more than 2 weeks after postchemotherapy FDG-PET, primary tumour was resected in all patients. Favourable FDG-PET response was concordant with favourable histological response in 58% and 68% of patients, when either postchemotherapy SUV < 2 or SUV ratio < 0.5 was considered, respectively. Absence of metastatic disease at baseline scan, favourable histological response, and postchemotherapy SUV < 2.5 were associated with longer progression-free survival, but statistical significance (*P* < 0.05) was not reached when metastatic patients were excluded. 

Gaston et al. [36] reviewed 12 ES and 19 OS patients treated with neoadjuvant chemotherapy and surgical resection, to assess whether different biological characteristics and response to chemotherapy of these tumours were related to their imaging characteristics on FDG-PET or FDG-PET/CT. Maximum and average SUV, SUV ratio, MTV, and percent of injected dose retained within the lesion (%ID) were obtained for the primary lesion on both baseline and posttherapy scan. A strong correlation was found between posttherapy SUV values lower than 2.5 and good histological response in OS patients, rather than in ES patients. Moreover, a 50% (or more) MTV reduction and a 70% (or more) ID reduction of the baseline value were associated with a favourable histological response in OS patients, while statistical significance in ES patients was reached for a reduction of at least 90% of baseline MTV value. ES responder patients showed a greater decrease in MTV and lower posttherapy MTV values in comparison with OS responders: this difference was probably due to the larger soft tissue component associated with ES, compared to OS, and therefore a greater propensity for early morphological regression. 

Kim et al. [11] retrospectively evaluated baseline and posttherapy FDG-PET scans in 23 paediatric patients with extremity or axial bone sarcomas (13 OS, 10 ES), to correlate metabolic changes with histological response (extent of tumoral necrosis). Higher posttherapy SUV values and lower decrease in SUV from baseline to posttherapy scan were observed in OS patients, in comparison with ES patients. All ES patients showed complete histological response, while 53% of OS patients were nonresponders. Lower posttherapy SUV values and higher SUV reduction from baseline were associated with favourable histological response in OS patients. Particularly, posttherapy SUV < 2.5 and a SUV reduction of at least 50% from baseline value were observed in 75–83% of responder patients.

Finally, London et al. [37] compared FDG-PET/CT with conventional imaging modalities, such as MRI, in the detection of paediatric primary bone lesions, prediction of their response to chemotherapy, and assessment of physeal involvement. Eight children (5 OS, 3 ES) with available histological diagnosis, baseline and postchemotherapy FDG-PET/CT and MRI examination were studied to evaluate response to therapy. In 6 patients with good response (5 OS patients), a greater reduction in maximum SUV was observed, in comparison with 2 patients with poor response. Moreover, baseline maximum SUV tended to be higher in lesions which showed better response after chemotherapy. Changes in size of the primary tumour did not significantly differ between patients with good response and patients with poor response, instead: both lesions with poor response and three ones with good response reduced in size.

## 4. General Remarks

As stated by our paper, many authors have investigated, prospectively or retrospectively, the role of FDG-PET and FDG-PET/CT in the assessment of response to neoadjuvant therapy in patients with OS and other kinds of primary bone sarcomas. In most cases, the degree of tumour necrosis on resected specimens was used as reference standard to evaluate the ability of FDG-PET or PET/CT to predict the presence of complete/good response after neoadjuvant chemotherapy or radiation therapy. According to Salzer-Kuntschick's six-grade scale [38], presence of <10% viable cells (grades I–III) was considered a good response, while more than 10% viable cells (grades IV–VI) was regarded as a poor response. Histological necrosis was mostly associated with the degree of reduction of FDG uptake within the primary tumour from baseline to postchemotherapy PET scan: a greater reduction correlated with extensive necrosis and, therefore, good response.

In almost all discussed studies, baseline and posttherapy maximum SUV, and their percent ratio, were used as a semiquantitative measurement of metabolic activity. However, maximum SUV only reflects the most active pixel within the lesion; in the presence of a wide necrotic area and a small amount of viable tissue with high FDG uptake, the percent degree of reduction of maximum SUV from baseline to posttherapy examination may be not significant (and therefore the patient considered as nonresponder), despite histological extensive necrosis. Moreover, maximum SUV values after therapy may exhibit a high variability and do not always correlate with histological response: high FDG uptake after therapy may be either ascribed to persistence of viable tumoral cells or to the presence of inflammation and reactive fibrosis in sites of tumoral response, and therefore associated with good response and favourable outcome [25]. Besides, baseline maximum SUV was not a useful predictor of metabolic and histological response; in one case [22] low FDG uptake at baseline has interfered with the detection of complete metabolic response and the patient was incorrectly classified as nonresponder despite extensive histological necrosis.

In most papers [17, 22–25, 28, 31, 33, 34, 36, 37], a comparison between FDG-PET or PET/CT and conventional diagnostic techniques, such as CT, MRI, and bone scintigraphy, was performed. Overall, the decrease in glycolytic activity, as measured by posttherapy FDG-PET or PET/CT, better correlated with histological necrosis, rather than the reduction in lesion size as assessed by posttherapy CT and/or MRI. Moreover, changes in T2 signal intensity within the lesion and reduction in surrounding soft tissue involvement did not significantly correlate with either histological necrosis or with progression-free and overall survival. Besides, posttherapy patterns of contrast enhancement within the suspected lesion, seen on both CT and MRI examinations, were not specific in discriminating between responder and nonresponder patients. A comparison between FDG-PET/CT and bone scintigraphy [28] showed that eventual changes in the osteotropic radiotracer uptake after treatment were not related to histological necrosis and posttreatment outcome, although respective pretreatment scans did not show any significant differences in radiotracer uptake: indeed, increased uptake on bone scintigraphy may reflect the healing response of bone in a site of successful treatment of lesions, rather than the presence of viable tumoral cells, as FDG-PET/CT does.

Most studies also assessed FDG uptake with measurement tools other than SUV, such as MTV, TLG, %ID, TBR, and their ratios. MTV has proved to be a reliable measurement of glycolytic activity within the lesion, showing a good correlation with tumour volume as measured by MRI and a much stronger association with histological response [26, 36]. TBR (obtained by drawing identical regions of interest over the tumour and the contralateral normal site) and TBR ratio (posttherapy TBR/baseline TBR) showed a high correlation with histological necrosis, in some cases much more significant than SUV [17–19]. However, Nair et al. [27] observed high variability in TBR ratio among good responder patients and no significant differences between responders and nonresponders. They hypothesized that increased FDG uptake in the site of a healing normal bone after successful treatment may have interfered with metabolic assessment of response. A segmental delayed scan (dual-phase FDG-PET or PET/CT), performed at least 3 hours after radiotracer injection, could be considered in order to overcome this issue, since a prolonged FDG uptake is less common in inflammatory sites than in residual malignant cells [39]. It has been hypothesized that high glycolytic activity (overexpression of transmembrane glucose transporter protein-1 and hexokinase-II) and underexpression of glucose-6-phosphatase in neoplastic cells could explain delayed increase in FDG uptake [39–41]. 

Some papers have also compared the assessment of metabolic response with FDG-PET between OS and ES patients [11, 23, 29, 36]: OS patients exhibited higher FDG uptake in posttherapy scan and lower percent reduction in maximum SUV, in comparison with ES patients, although all responder patients, both ES and OS, usually had maximum SUV after therapy lower than at baseline. Furthermore, metabolic response better correlated with histological necrosis and overall survival in OS patients, with significantly greater maximum SUV reduction rates in responders. ES patients usually exhibited a larger reduction in lesion volume, probably because of the larger soft tissue component associated with ES, compared to OS, and therefore a greater propensity for early morphological regression; anyhow, no significant correlation was found between reduction in lesion volume and overall survival in both ES and OS patients. 

## 5. Conclusion

Finally, despite methodological heterogeneity in the included studies, FDG-PET or PET/CT seems to be sensitive and reliable diagnostic tools in the assessment of metabolic response in patients with OS, after baseline PET evaluation has been performed in advance. Moreover, in most patients, good metabolic response better correlates with favourable histological outcome, high progression-free and overall survival rate, rather than reduction in lesion volume. Possible inflammation and reactive fibrosis in sites of tumoral response should be considered as a source of false positive findings.

## Figures and Tables

**Table 1:** Prospective studies.

Authors (reference)	Journal/year	Patients (OS)	Gender (% male)	Age (range)	Quantification indexes
Jones et al. [17]	J Nucl Med/1996	9 (3)	67	15–65	SUV, TBR
Schulte et al. [18]	J Nucl Med/1999	27 (27)	63	5–33	%TBR
Ye et al. [19]	Ann Nucl Med/2008	15 (15)	60	7–33	SUV-ratio, %TBR
Sato et al. [20]	Clin Exp Metastasis/2008	13 (13)	77	11–54	SUV
Benz et al. [21]	J Nucl Med/2008	33 (8)	49	19–86	SUV, TBR, SUV-ratio, %TBR
Benz et al. [22]	Sarcoma/2010	12 (6)	42	18–61	SUV, SUV-ratio
Denecke et al. [23]	Eur J Nucl Med Mol Imaging/2010	27 (11)	56	3–18	SUV, SUV-ratio
Tateishi et al. [24]	Clin Nucl Med/2011	42 (NR)	62	32–72	SUV
Bajpai et al. [25]	J Pediatr Hematol Oncol/2011	31 (31)	81	5–66	SUV, SUV-ratio, MB
Im et al. [26]	Eur J Nucl Med Mol Imaging/2012	20 (20)	50	10–25	SUV, MTV, TLG, SUV-ratio, MTV-ratio, %TLG

SUV: standardized uptake value; TBR: tumour-to-background ratio; MB: metabolic burden; TLG: total lesion glycolysis; %TBR: percent change in TBR (baseline to posttherapy); %TLG: percent change in TLG (baseline to posttherapy); NR: not reported.

**Table 2:** Retrospective studies.

Authors (reference)	Journal/year	Patients (OS)	Gender (% male)	Age (range)	Quantification indexes
Nair et al. [27]	Clin Positron Imaging/2000	16 (16)	47	15–29	TBR, %TBR
Franzius et al. [28]	Clin Nucl Med/2000	17 (11)	76	5–36	T : NT-ratio
Hawkins et al. [29]	Cancer/2002	33 (18)	67	6–19	SUV, SUV-ratio
Iagaru et al. [30]	Clin Nucl Med/2008	14 (7)	57	18–56	SUV, SUV-ratio
Mahajan et al. [31]	Pediatr Blood Cancer/2008	39 (39)	49	6–20	SUV
Costelloe et al. [32]	J Nucl Med/2009	31 (31)	61	9–65	SUV, TLG, %TLG
Piperkova et al. [33]	Clin Nucl Med/2009	83 (NR)	NR	NR	SUV
Hamada et al. [34]	Ann Nucl Med/2009	11 (11)	64	10–68	SUV, SUV-ratio
Hawkins et al. [35]	Cancer/2009	40 (40)	NR	7–31	SUV, SUV-ratio
Gaston et al. [36]	Skeletal Radiol/2011	31 (19)	42	14–38	SUV, SUV-ratio, MTV, %ID
Kim et al. [11]	Cancer Res Treat/2011	23 (13)	69	3–19	SUV, SUV-ratio
London et al. [37]	Pediatr Radiol/2012	8 (5)	NR	NR	SUV

SUV: standardized uptake value; T: NT-ratio: tumour to nontumour ratio; TBR: tumour-to-background ratio; TLG: total lesion glycolysis; %TBR: percent change in TBR (baseline to posttherapy); %TLG: percent change in TLG (baseline to posttherapy); MTV: metabolic tumour volume; %ID: percent injected dose retained within the lesion; NR: not reported.
